# Genetic background influences adaptation to cardiac hypertrophy and Ca^2+^ handling gene expression

**DOI:** 10.3389/fphys.2013.00011

**Published:** 2013-03-06

**Authors:** Steve B. Waters, Douglass M. Diak, Matthew Zuckermann, Paul H. Goldspink, Lara Leoni, Brian B. Roman

**Affiliations:** ^1^Department of Radiology, The University of ChicagoChicago, IL, USA; ^2^Department of Physiology, Medical College of WisconsinMilwaukee, WI, USA

**Keywords:** mouse, heart, hypertrophy, Ca^2+^-handling, gene expression, hemodynamics

## Abstract

Genetic variability has a profound effect on the development of cardiac hypertrophy in response to stress. Consequently, using a variety of inbred mouse strains with known genetic profiles may be powerful models for studying the response to cardiovascular stress. To explore this approach we looked at male C57BL/6J and 129/SvJ mice. Hemodynamic analyses of left ventricular pressures (LVPs) indicated significant differences in 129/SvJ and C57BL/6J mice that implied altered Ca^2+^ handling. Specifically, 129/SvJ mice demonstrated reduced rates of relaxation and insensitivity to dobutamine (Db). We hypothesized that altered expression of genes controlling the influx and efflux of Ca^2+^ from the sarcoplasmic reticulum (SR) was responsible and investigated the expression of several genes involved in maintaining the intracellular and sarcoluminal Ca^2+^ concentration using quantitative real-time PCR analyses (qRT-PCR). We observed significant differences in baseline gene expression as well as different responses in expression to isoproterenol (ISO) challenge. In untreated control animals, 129/SvJ mice expressed 1.68× more ryanodine receptor 2(Ryr2) mRNA than C57BL/6J mice but only 0.37× as much calsequestrin 2 (Casq2). After treatment with ISO, sarco(endo)plasmic reticulum Ca^2+^-ATPase(Serca2) expression was reduced nearly two-fold in 129/SvJ while expression in C57BL/6J was stable. Interestingly, β (1) adrenergic receptor(Adrb1) expression was lower in 129/SvJ compared to C57BL/6J at baseline and lower in both strains after treatment. Metabolically, the brain isoform of creatine kinase (Ckb) was up-regulated in response to ISO in C57BL/6J but not in 129/SvJ. These data suggest that the two strains of mice regulate Ca^2+^ homeostasis via different mechanisms and may be useful in developing personalized therapies in human patients.

## Introduction

Heart disease by any measure is a significant and ever increasing health risk around the world. Cardiac pharmacogenetic studies have revealed that although pharmaceutical efficacy is high for a population, the range of individual response is great because of genetic variability of individuals within the population (Roden et al., 2011). This is particularly true for β-blockers and DNA variants are suspected to modulate these differences (Brodde et al., 2001; Port and Bristow, 2001). These types of variations are one of the motivators for pursuing theranostic “personalized medicine.” As individual variation impacts drug efficacy, consideration should be given to how genetic variation extends to the inherent development and progression of cardiac disease. While advances in biology have focused heavily on the impact of a specific gene on the outcome of a disease, taking a candidate gene approach has not been entirely productive. Frequently at fault in animal models is the effect of the selected genetic background, which can cause transgenes to produce different and even contradictory results (Davie et al., 2007; Cook et al., 2009). Yet moving to a whole genome approach is an extremely difficult and exhaustive pursuit. To further compound the problem, transgenic mice are frequently created on one genetic background and then insufficiently backcrossed to another. As such, there is a growing appreciation for variation in the physiology between different strains. While genotypes are important, it is the functional phenotype that is clinically observed that ultimately matters. To investigate differences in cardiac function we took a systems biology approach to analyze two common strains, C57BL/6J mice and 129/SvJ mice during hypertrophic stress. Both strains are commonly used to generate transgenic animals and have previously observed significant morphological differences (Grubb et al., 2009), such as the myocytes of 129/SvJ mice are smaller, more tightly packed and have a decreased t-tubule density (Shah et al., 2010). Additionally, 129/SvJ mice carry two rennin genes (Ren 1^d^ and Ren-2) which is likely reflected in the observed elevated blood pressure (Wang et al., 2002; Lum et al., 2004).

The focus of this work is the impact of background genetics on cardiac function and the response to a hypertrophic stimulus. We constrained our investigation further to the integral role of calcium handling via examination of gene expression differences between two strains as a basis for a differential hypertrophic response. It is well-established that the production of cytotoxicity via Ca^2+^ overload is a major stimulus to pathologic hypertrophy in model systems. In the intact failing human heart, it appears that β 1-adrenergic signaling accounts for the majority of pathologic hypertrophy in chronic heart failure (Port and Bristow, 2001). This key role in heart failure made β-adrenergic signaling prime candidates for comparison in gene expression and *in situ* hemodynamics. Additionally, we took the next step and investigated changes in the expression of several important genes that regulate the sequestering of calcium in the sarcoplasmic reticulum (SR). The ryanodine receptor complex controls the efflux of calcium from the SR upon the induction of calcium-induced calcium release (CICR) (Kunitomo and Terentyev, 2011). As such, we analyzed the expression of the ryanodine receptor (Ryr2) and its associated proteins triadin (Trdn), junctate/junctin (Asph). Calsequestrin 2 (Casq2) was included as it is the most abundant protein located in the SR, acting both as a Ca^2+^ sink and coordinating release of Ca^2+^ through the Ryr2 channel (MacLennan and Wong, 1971; Periasamy and Huke, 2001). In addition, we quantified the mRNA levels for sarcoplasmic reticulum calcium ATPase (Serca2) and its associated protein phospholamban (Pln) as these proteins actively pump intracellular calcium back into the SR to facilitate muscle relaxation. Finally, creatine kinase muscle and brain (Ckm and Ckb) isoforms play an important role in maintaining cellular energetics that are necessary for pumping calcium and were also examined. The expression of these genes was quantified under both control and isoproterenol (ISO) induced hypertrophic conditions in conjunction with *in situ* hemodynamics. Our results show differences in the expression of genes between these two strains and suggest different mechanisms by which the two strains maintain calcium equilibrium between the cytosol and SR. Utilizing multiple murine strains as potential surrogates for modeling variation in the human genotype may ultimately be useful in the development of pharmaceutical agents and scoring response to therapeutic regimens.

## Methods

### Animal care

Male C57BL/6J and 129/SvJ mice (8–13 weeks old) were obtained from Harlan Laboratories, housed in the University of Chicago Animal Resource Center under standard housing and diet conditions. Experimental protocols were approved by the University of Chicago Institutional Animal Care and Use Committee (IACUC).

### Pharmaceuticals

Hypertrophy was induced by chronic ISO (Sigma) treatment (S.C. 47 mg/kg BW) via Alzet micro-osmotic pumps Model 1003D (Durect Corp) for 3 days. Pumps were removed 12 h prior to conducting any experiments on the treated or sham animals. Control animals received pumps with vehicle only and were otherwise treated identically. Dobutamine (Db) (Sigma) was infused at 8.3 μg/min/gBW for 10 min and 83 μg/min/gBW for 1 min in both control and hypertrophic animals to increase contractility.

### Hemodynamic measurements

Mice were initially anesthetized with 2–3% isoflurane in 100% oxygen in an induction box and then maintained under anesthesia through surgery with 1–2% isoflurane as needed delivered through a mask. A Millar microtip pressure catheter was inserted through the right carotid and advanced to the left ventricle (LV) of the mouse heart to record left ventricular pressure (LVP), the first derivative (±dP/dt), and heart rate (HR).

### RNA analysis

Hearts from C57BL/6J and 129/SvJ male mice were surgically removed 6 h after the removal of the pumps. The apex was excised for RNA isolation using Trizol reagent (Invitrogen). RNA was checked for purity via UV spectroscopy and integrity via agarose gel analysis. Samples with a degraded 28S band were excluded from further analyses. RNA was diluted to 25 ng/ul for qRT-PCR experiments. Genes of interest were PCR cloned using TOPO-TA cloning kit (Invitrogen) and subsequently used as template for RNA runoffs. This template RNA was DNase (Invitrogen) treated, ethanol precipitated, and diluted serially to generate standard curves for qRT-PCR applications. Primers for template and qRT PCR are listed in Table 1. qRT-PCR experiments were conducted with 100 ng RNA in 25 μl reactions using SYBER Green 2× Reaction mix on a MyIQ single color detection system (Bio-Rad). β-actin mRNA was quantified and found to be statistically indistinguishable between the four groups of animals. Data is reported as fold expression relative to untreated C57BL/6J animals. Due to recurrent non-specific amplification, primers for AdBR1 and AdBR2 were obtained from SABiosciences (PPM05035A-200 and PPM04265B-200). Results for AdBR1 and AdBR2 are reported as normalized fold expression to β-actin as determined using IQ5 software (Bio-rad).

**Table 1:** **Gene and associated qRT primers**.

**Gene**	**Accession number**	**qRT primers**	**qRT primer Tm**	**Template primers**
Ryr2	NM_023868	CAGCATCTCGTTTCGCATTA	58.7	TGTGAATCATGTCAGCAGCA
		GGCTGTGTTCCACCTTCAAT		CAATGCCAGCAAAGTCTTGA
Casq2	NM_009814	ATTTATGGATGAGCCCACG	57.0	TACACAGCTGCAGGACCAAG
		GTCACTCTTCTCCGCAAGG		CAATGCCCACACATTTCAAG
Asph	Consensus	TGGGAGAAGAGGAGGGATTT	61.0	TGGCAGTACTGGTAGCCACA
		TGGCATCATCCACATCAAAG		CCCTTCCCTCTATCCTCCTG
Trdn	NM_029726	GATGATGGCAAAAGAGGACAA	57.0	TCAGCCAGAAAATCCAGGAA
		CTTCTTTCTGGCCTTTGGTG		CAGCTGGCATCTCTTTGTCA
Serca2	NM_001110140	TGGGCAAAGTGTATCGACAG	58.7	TGGAGAACGCTCACACAAAG
		GGTCAGGGACAGGGTCAGTA		CAGTGGGTTGTCATGAGTGG
Pln	NM_001141927	CACTGTGACGATCACCGAAG	55.0	CTCCCATAAACCTGGGAACA
		ATAGCCGAGCGAGTGAGGTA		AGGGGACAACCACTTCCTCT
Ckm	NM_007710	GAGATTCTCACTCGCCTTCG	58.7	ATGCCGTTCGGCAACACC
		GCCCTTTTCCAGCTTCTTCT		CTACTTCTGCGCGGGGAT
Ckb	NM_021273	AAGTTCTCGGAGGTGCTCAA	55.0	GAGATCCTCGAGATGCCCTTCTCCAA
		CCGTTGCTCCATCTCAATG		GAGATCGTCGACTCACTTCTGGGCCG
Actb	NM_007393	GCTCTTTTCCAGCCTTCCTT	55.8	TGTTACCAACTGGGACGACA
		CTTCTGCATCCTGTCAGCAA		AGGGAGACCAAAGCCTTCAT

### Statistical analysis

Data (mean ± SE) are presented as relative fold expression to untreated C57BL/6J animals. Data was analyzed on SigmaStat analysis software (Systat Software, Inc.) using the Two-Way ANOVA test and the Holm–Sidak *post-hoc* pairwise procedure. Significant changes between groups are indicated in the figures by asterisks.

## Results

### Morphology

**Figure 1:**
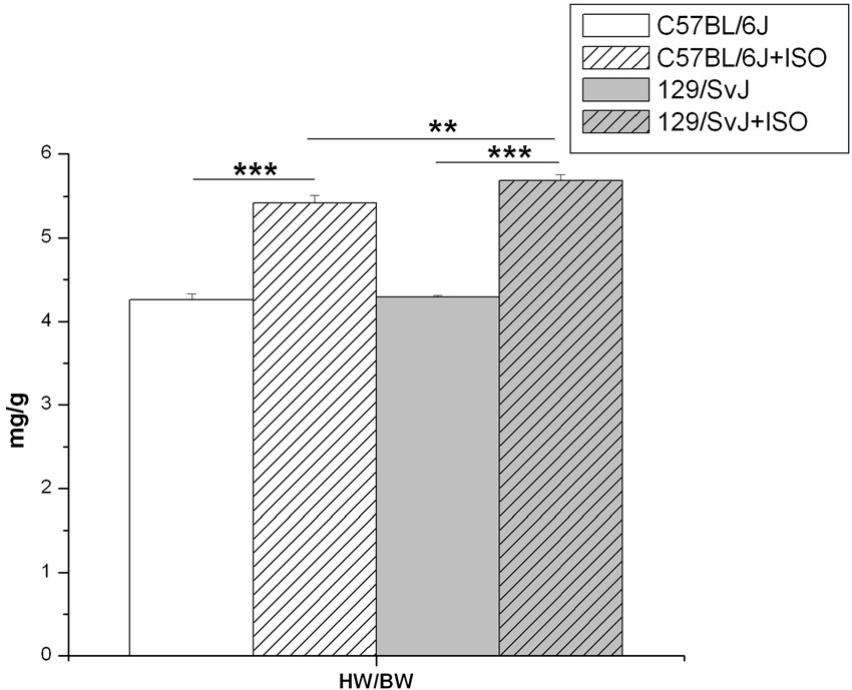
**Isoproterenol induced hypertrophy in C57BL/6J and 129/SvJ mice.** Heart weight/Body weight ratios were determined and are reported here as mg/g. ^**^P < 0.01; ^***^P < 0.001.

Animal body weights (Bw) were measured along with isolated hearts (Hw) after removal of major vessels and auricular appendages. The average control Hw/Bw was 4.26 ± 0.073 mg/g (*n* = 14) for C57BL/6J and 4.29 ± 0.021 mg/g (*n* = 15) for 129/SvJ. ISO significantly induced cardiac hypertrophy in C57BL/6J by 26% [Hw/Bw of 5.42 ± 0.09 mg/g (*n* = 10)] and significantly greater (32%) in 129/SvJ [5.69 ± 0.071 mg/g (*n* = 17)]. Results and significance are illustrated in Figure 1.

### Hemodynamics

**Figure 2:**
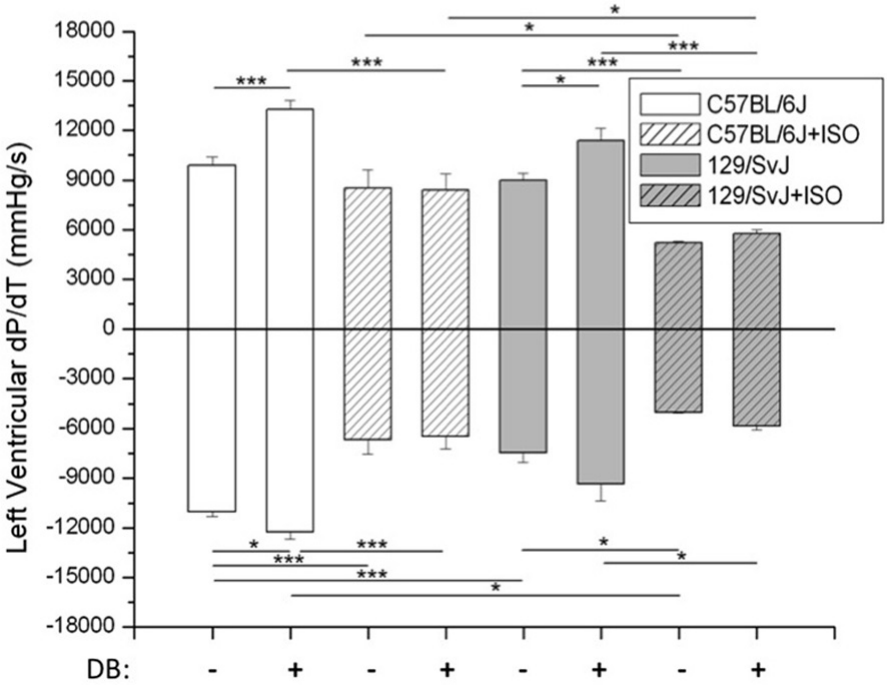
***In situ* hemodynamic analyses of 129/SvJ and C57BL/6J mice.** Differences in contractility (+dP/dt) and relaxation (−dP/dt) between strains with and without ISO treatment are reported. Asterisks indicate significance. ^*^P < 0.05; ^***^P < 0.001.

Hemodynamic measurements and significance are presented in Figure 2. In brief, at baseline both C57BL/6J and 129/SvJ strains functioned similarly although 129/SvJ demonstrated diminished relaxation [C57BL/6J: ±dP/dt 9893 ± 519/11047 ± 254 mmHg/s (*n* = 7); 129/SvJ: ±dP/dt 8978 ± 446/7442 ± 609 mmHg/s (*n* = 7)]. Db stimulation had the expected increase in contractility in both strains although −dP/dt did not reach significance in 129/SvJ [C57BL/6J: ±dP/dt: 13253 ± 559/12232 ± 446 (*n* = 7); 129/SvJ ±dP/dt: 11355 ± 750/9346 ± 1009 mmHg/s (*n* = 7)]. Hypertrophy reduced contractility and although C57BL/6J mice did not demonstrate a significant decrease in +dP/dt, there was a dramatic decrease in −dP/dt [C57bl/6J: ±dP/dt 8496 ± 1116/6685 ± 837 mmHg/s (*n* = 5)]. In contrast 129/SvJ mice demonstrated a dramatic decrease in overall contractility which was significantly more than C57BL/6J [129/SvJ ±dP/dt: 5214 ± 64/5013 ± 51 mmHg/s (*n* = 4)]. As expected neither C57BL/6J nor 129/SvJ hypertrophic mice responded significantly to Db stimulation [C57BL/6J ± dP/dt: 8391 ± 967/6478 ± 725 mmHg/s (*n* = 5)] 129/SvJ ± dP/dt: 5749 ± 229/5834 ± 263 mmHg/s (*n* = 4).

### Ryanodine channel expression

**Figure 3:**
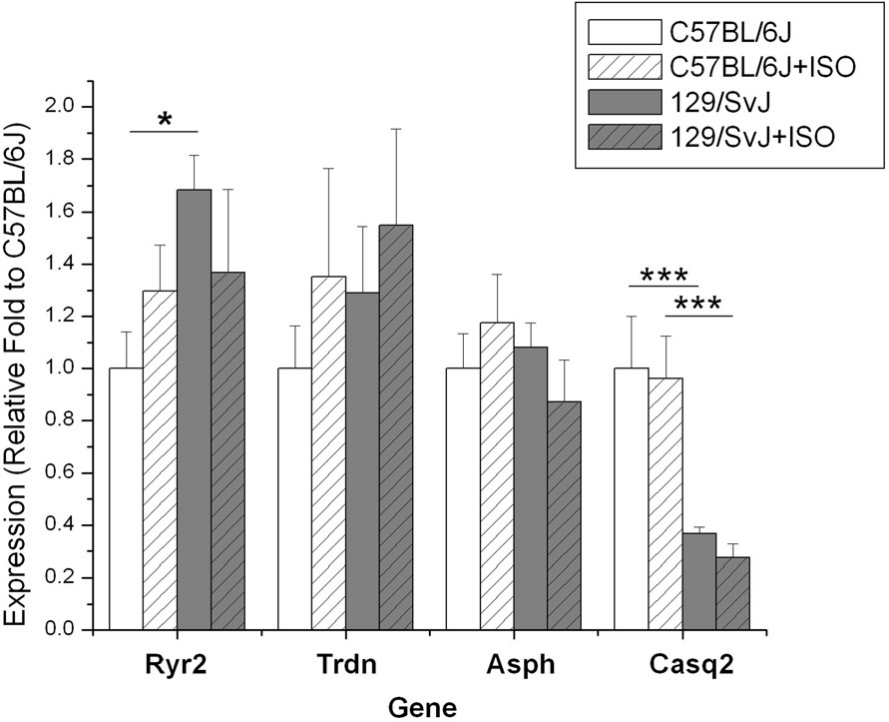
**Expression of genes in the ryanodine receptor complex is different between 129/SvJ and C57bl/6J mice.** Significant changes between groups are indicated by bars. *P*-values are indicated starred designation. ^*^P < 0.05; ^***^P < 0.001.

The expression of four genes (Ryr2, Casq2, Asph, and Trdn) associated with the ryanodine channel was quantified resulting in strain dependence. Ryanodine receptor expression was significantly higher in 129/SvJ animals [1.68 ± 0.131 (*n* = 11)] compared to C57BL/6J (1 ± 0.14, *n* = 11). In this complex there was no significant impact of ISO induced hypertrophy on gene expression but there was an opposite trend between the two strains. The result was the statistical equivalence of post-treatment Ryr2 expression 129/SvJ (1.37 ± 0.32, *n* = 11) and C57bl/6J (1.30 ± 0.16, *n* = 9). Casq2 expression was significantly impacted by strain and was dramatically lower in 129/SvJ animals (0.367 ± 0.026, *N* = 11) relative to C57BL/6J (1 ± 0.199, *N* = 11). Hypertrophy had no significant impact on Casq2 expression in either strain (C57BL/6J 0.96 ± 0.161, *n* = 11; 129/SvJ: 0.276 ± 0.052, *n* = 11). Gene expression of neither triadin nor junctate/junctin, both products of the aspartate β-hydroxylase (ASPH), was altered significantly by either strain or treatment (Triadin—C57BL/6J baseline: 1 ± 0.164; ISO: 1.35 ± 0.41; 129/SvJ baseline: 1.29 ± 0.25; ISO: 1.55 ± 0.37; β-hydroxylase (ASPH)—C57BL/6J baseline: 1 ± 0.13, ISO: 1.30 + 0.18, and 129/SvJ baseline: 1.08 ± 0.09, ISO 0.87 ± 0.16). Data and significance are summarized in Figure 3.

### Sarcoplasmic reticulum ATPase and phospholamban

**Figure 4:**
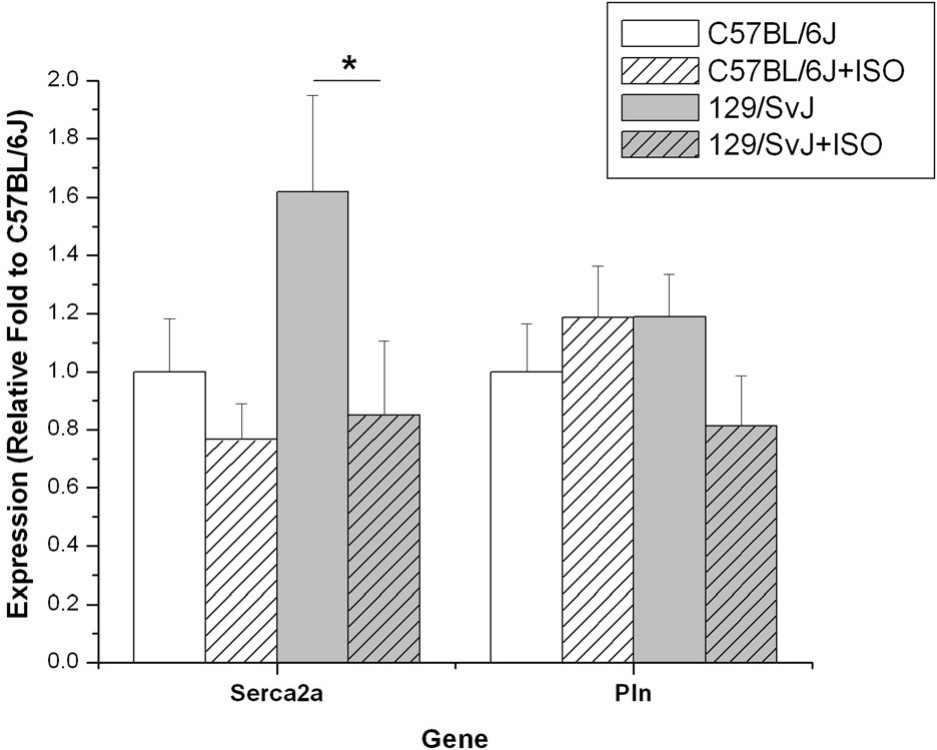
**Expression of Serca2 and Pln differ in 129/SvJ and C57BL/6J mice.** The Serca2 pump is elevated in 129/SvJ mice relative to C57BL/6J but is significantly down-regulated after ISO challenge. Pln is also down-regulated in 129/SvJ mice after ISO treatment. Significant changes are indicated by starred designation. ^*^P < 0.05.

Expression of Serca2 was significantly different between strains (C57BL/6J: 1.0 ± 0.18; 129/SvJ:1.62 ± 0.33) and was dramatically decreased by hypertrophy in 129/SvJ (0.85 ± 0.25) but not in C57BL/6J hearts (0.77 + 0.12). Pln expression was not significantly different due to strain (C57BL/6J:1 ± 0.16;0.129/SvJ:1.19 ± 0.15) and ISO-induced hypertrophy did not significantly impact expression in C57BL/6J (1.19 ± 0.18) or 129/SvJ (0.81 ± 0.17). Data and significance are summarized in Figure 4.

### Creatine kinase isoform expression

**Figure 5:**
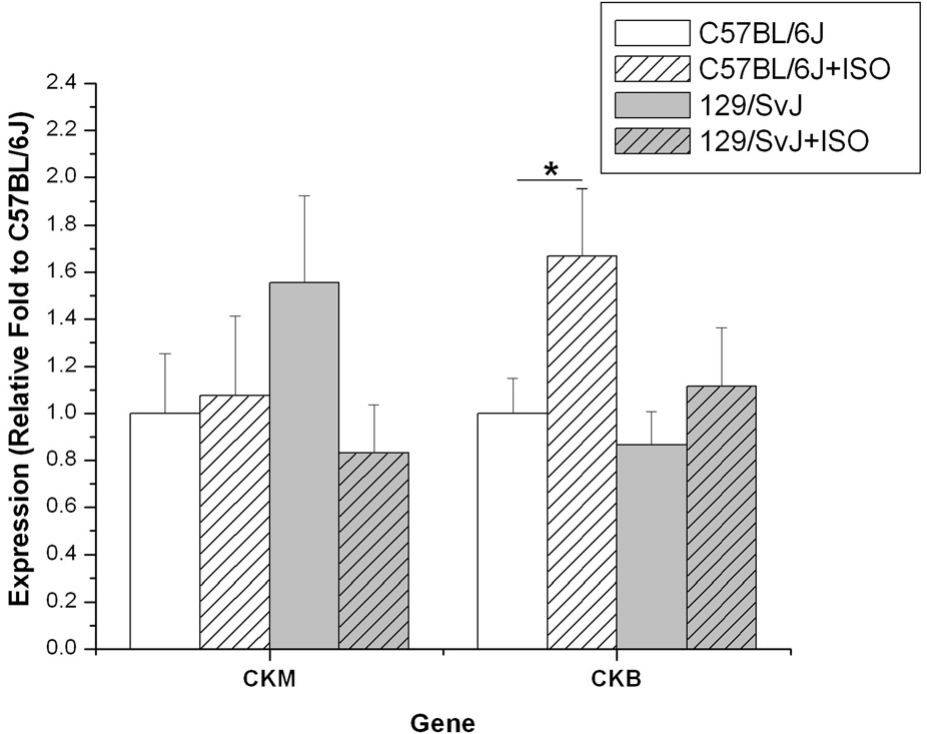
**Creatine kinase expression is elevated in the hearts of 129/SvJ mice.** Quantities of CK isoform mRNA are indicated in each graph. Expression of each isoform in 129/SvJ mice differs in the response to ISO when compared to C57BL/6J mice. Significant changes are indicated by starred designation. ^*^P < 0.05.

The expression of both brain and muscle isoforms of Ckb were assessed in cardiac tissues. Although there was a trend for CKM to be expressed at a lower level in C57BL/6J compared to 129/SvJ at baseline, it did not reach statistical significance (C57BL/6J: 1 ± 0.25; 129/SvJ:1.56 ± 0.37). ISO treatment had no impact on C57BL/6J (C57BL/6Jiso: 1.07 ± 0.34), but there was a striking trend in 129/SvJ indicating decreased expression although it did not reach statistical significance (129/SvJiso: 0.83 ± 0.20). In contrast, CKB expression was elevated in iso-treated C57BL/6J animals (1.67 ± 0.28) relative to baseline (C57BL/6J: 1 ± 0.15). There was no significant difference in 129/SvJ control (0.86 ± 0.14) or treated (1.12 ± 0.25) groups. Results and significance are summarized in Figure 5.

### Beta-adrenergic receptor expression

**Figure 6:**
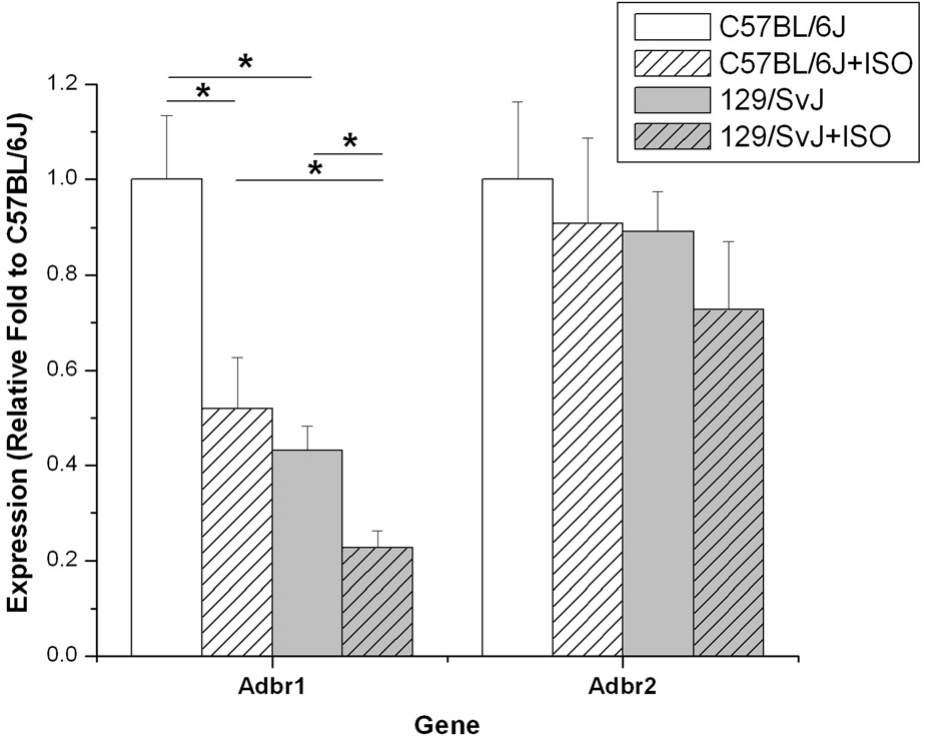
**Adbr1 is down regulated in 129/SvJ mice.** Expression of the β 1-Adrenergic receptor in 129/SvJ mice is reduced 2-fold relative to C57BL/6J. Both strains showed 2-fold reductions in Adbr1 expression after ISO challenge indicating an appropriate response to the β 1 agonist. Significant changes are indicated by starred designation. ^*^P < 0.05.

Relative fold expression of the β1 and β2 adrenergic receptors was determined in all four groups of mice. Consistent with previous reports, Adbr1 was the predominant receptor expressed in cardiac tissue (data not shown) (Brodde et al., 2001). Adbr1 expression was strain dependent being over 2-fold higher in control C57BL/6J (1 ± 0.134) compared to 129/SvJ (0.43 ± 0.05). Treatment with ISO caused the down-regulation of Adbr1 in both C57BL/6J (0.52 ± 0.11) and 129/SvJ (0.23 ± 0.04) mice by approximately 2-fold. Adbr2 expression was not significantly different between any of the four groups. Data for relative expression and significance are represented in Figure 6.

## Discussion

Recent studies have shown that different strains of mice present characteristic variations in cardiac physiology (Shah et al., 2010). For example, hearts of 129/SvJ and C57BL/6J mice differ not only morphologically but also in Ca^2+^ homeostasis (Barrick et al., 2007; Shah et al., 2010). Consistent with these reports, our hemodynamic analyses indicate altered Ca^2+^ homeostasis and a blunted response to β-adrenergic agonists in 129/SvJ animals may be the basis for a greater hypertrophic response. These two strains also show different sensitivities and altered responses depending on the type of cardiovascular challenge. For example, C57BL/6J mice have been reported to be more sensitive to aortic constriction than 129/SvJ mice (Barrick et al., 2007). In our study, 129/SvJ mice demonstrated an increased sensitivity to ISO-induced hypertrophy compared to C57BL/6J mice, by developing significantly more mass (Figure 1).

A prodigious amount of energy is spent maintaining the calcium gradient in the SR, in which intra-sarcolemmal concentrations exceed cytoplasmic concentrations by 5000-fold (Barry and Bridge, 1993). Conceptually, this gradient is modulated through three mechanisms, passive retention by calcium binding proteins in the SR, escape through the ryanodine receptor complex, and active pumping of Ca^2+^ into the SR via SERCA. As such, it is easy to envision that alterations in the capacity of any of the mechanisms can be offset by concomitant changes in the other two. The balance of these three mechanisms is likely to impact the cardiac physiology of the organism. Consequently, we investigated the expression of proteins controlling Ca^2+^ flux from the SR. Based on the idea that each strain differs mechanistically in maintaining Ca^2+^ homeostasis, perhaps they are predisposed to different cardiovascular outcomes following stress. For example, based on their transcriptional profile, ISO-induced cardiac hypertrophy in C57BL/6J mice has been shown to resemble ischemic-related human cardiac hypertrophy (Galindo et al., 2009) due to alteration in several calcium homeostatic and handling related genes.

Interestingly, we observed over 2-fold higher expression of calsequestrin in the hearts of C57BL/6J animals. Calsequestrin has long been considered to act as a calcium sink in the SR (Campbell et al., 1983). Using adenoviral technology in adult rat cardiomyocytes, Terentyev et al. demonstrated that the amount of CASQ2 expression correlated with both calcium storage in the SR as well as the size and duration of calcium transients upon stimulation (Terentyev et al., 2003). Similarly, transgenic mice over-expressing Casq2 have elevated SR Ca^2+^ content (Jones et al., 1998). Based on these data one would predict C57BL/6J mice to have elevated CASQ2 protein and SR Ca^2+^ content compared to 129/SvJ mice. This is, however, in contrast to published observations in which 129/SvJ mice had the highest SR Ca^2+^ content of four strains assayed (Shah et al., 2010) indicating the importance of other mechanisms of calcium sequestration. In fact, mice with ablated CASQ2 were able to maintain normal SR calcium content albeit with a 50% increase in SR volume (Knollmann et al., 2006). This points to the need for further characterization of these genes at the protein level including post-translational modifications as expression does not always correlate with neither protein quantity nor activity.

In addition to the role CASQ2 plays in binding calcium, there is mounting evidence of a critical role in mediating the response of ryanodine receptor to SR luminal calcium concentrations (Terentyev et al., 2008; Knollmann, 2009) where CASQ2 can act as a sensor by adopting a more compact configuration when binding Ca (Cozens and Reithmeier, 1984). Interactions with TRDN and JNC are also clearly involved as shown in CASQ2 knockout mice. These nulls presented a dramatic reduction in both TRDN and JNC, most likely due to decreased protein stability and/or decreased protein synthesis, as expression of both genes was unchanged (Knollmann et al., 2006). Similarly, higher concentrations of CASQ2 would also impact not only the physical conformation of CASQ2 but its intermolecular interactions with junctate, junctin, triadin as well as with other CASQ2 molecules. In fact, over-expression of calsequestrin in FVB mice led to increased Ca^2+^ storage capacity of the SR. However, these Ca^2+^ ions are not available for release during contraction causing a depression in contractile function and ultimately cardiac hypertrophy (Sato et al., 2003).

Given their significantly higher Casq2 levels, a similar behavior would be expected of C57BL/6J mice when compared to 129/SvJ. On the contrary, they exhibited a more muted response to chronic ISO stimulation. This could be explained by considering that in addition to the high capacity binding loci with elevated Casq2, lower Ryr2 expression was observed in C57BL/6J mice, resulting in a dramatically different ratio of Ryr2:Casq2 in C57BL/6J vs. 129/SvJ. We believe that this may impact the efflux of Ca^2+^ from the SR by altering the interactions among the proteins in the ryanodine complex. Based on the current data, there is insufficient information to determine the mechanism and whether there is mediation through triadin and junctate. The physical restraints of fewer points of efflux coupled with higher buffering capacity suggest an environment overall better suited for calcium retention. It is conceivable that passive calcium retention is energetically favorable and may justify the ISO-induced attenuated hypertrophic response in C57BL/6J mice since β-adrenergic stimulation is reflected in greater calcium efflux through phosphorylation of both Ryr2 and Pln (Li et al., 2000; Shan et al., 2010). Including both strains in future studies will provide useful insights into further defining the roles of TRDN and JNC in regulating luminal SR calcium concentration.

Although requiring further examination, the attrition of 129/SvJ mice was greater during the ISO treatment compared to C57BL/6J. This may be related to catecholamine-induced polymorphic ventricular tachycardia (CPVT) in which adrenergic stimulation can frequently cause lethal arrhythmias in mice with CASQ2 loss of function alterations (Song et al., 2007; Terentyev et al., 2008). Mutations associated with familial CPVT are frequently found in the CASQ2 and RYR2 genes. We would propose that fewer Ryr2 channels would reduce the likelihood of a spontaneous depolarization and explains why we do not observe similar arrhythmias in C57BL/6J animals. We believe that similar variations in the human population can exist and that finding such populations will allow for better prevention of disease and, ultimately, more effective therapeutic intervention when necessary.

While we consider the luminal retention of Ca^2+^ by CASQ2 to be very important, other factors need to be considered. It is our hypothesis that adaptations in 129/SvJ SR compensate for reduced CASQ2 expression. Higher Serca2 levels in 129/SvJ mice indicate that this strain relies more heavily on active translocation of cytosolic calcium to the SR lumen. This burden may create a chronic strain on cellular ATP pools in 129/SvJ mice. This is reflected in the down-regulation of β-receptors we observe in 129/SvJ mice. The higher flux of calcium upon β-adrenergic stimulation would theoretically have a greater impact on 129/SvJ mice by increasing the energetic demands of maintaining appropriate calcium concentration gradients. Chronic β-adrenergic stimulation affects Serca2 activity by phosphorylating and dissociating its inhibitor Pln. This likely explains the change of Serca2 with ISO treatment in 129/SvJ mice. Rat hearts show decreased Pln (Linck et al., 1998) and higher Pln phosphorylation after ISO (Bossuyt et al., 2005), both having the effect of increasing Serca2 activity. This effect, however, is not found in most of patients presenting heart failure. Rather, Serca2 expression has been found to be greatly reduced in several cardiac conditions, including hypertrophic cardiomyopathies. Therefore, one must carefully choose the type of stress but equally carefully the genetic background which more closely recapitulate the related human condition.

Genetic variation is well-known to play a role in the pathophysiology of human left ventricular hypertrophy and in cardiac failure overall. Most studies have focused on identifying the role of polymorphisms and genetic variants of single candidate genes, with inconsistent result (Bleumink et al., 2004). β-adrenergic receptor blockers can improve symptoms and mortality rates in heart failure, although with significant interindividual variations (Liggett, 2000). Polymorphisms have been linked with these variations; however, here we have demonstrated that cardiac failure involves a complex system of genes and their reciprocal interactions. We believe these results have multiple implications. They can find clinical application in helping predict the risk for heart failure and characterize individual sensitivities to drugs, therefore driving personalized treatment.

Clinical studies have indicated different geometric patterns of LV hypertrophy exist in patients during the development and management of hypertension. Patients with concentric, eccentric LV hypertrophy, and normal baseline geometry were identified and appeared fairly stable without transition to dilatation over the 5 years followed (Conrady et al., 2005). Consequently, it is not known whether the remodeling observed represents different temporal stages in the development of heart failure or whether there is a genetic predisposition in some individuals. Further exploration and comparison of inbred strain-specific responses to hypertrophic stimuli could lead to better preclinical models for partitioning LVH by LV geometry which could provide prognostic value in stratifying patients based on cardiovascular risk.

Pharmacogenetics studies and investigations of cardiomyopathies must keep the strain effect into consideration and different mice strains can and should be used. Because it is ultimately too expensive to create transgenic animals on a large number of genetic backgrounds, it becomes very important to choose an appropriate one. Through proper selection of the correct strains, their crossing and the F1 hybrids can help determine the impact of a transgene on a divergent enough background to better determine the effects of that gene in a whole environment rather than in isolation. Further experiments including altering the hypertrophic stress are predicted to result in different adaptations (Galindo et al., 2009). Expanding the number of strains is likely to shed further light on gene interactions not fully appreciated and similar to what is found in the genetically larger human population.

## Author contributions

Steve B. Waters, Douglass M. Diak, Matthew Zuckermann, and Brian B. Roman performed experiments. Steve B. Waters, Lara Leoni, and Brian B. Roman drafted manuscript. Douglass M. Diak and Steve B. Waters prepared figures. Douglass M. Diak, Steve B. Waters, and Brian B. Roman analyzed data. Steve B. Waters, Douglass M. Diak, Brian B. Roman interpreted results of experiments. Steve B. Waters, Lara Leoni, Paul H. Goldspink, and Brian B. Roman revised manuscript. Steve B. Waters, Lara Leoni, and Brian B. Roman approved final version of manuscript. Paul H. Goldspink and Brian B. Roman conception and design of research.

### Conflict of interest statement

The authors declare that the research was conducted in the absence of any commercial or financial relationships that could be construed as a potential conflict of interest.
